# Screening and Bioinformatics Analyses of Key miRNAs Associated with Toll-like Receptor Activation in Gastric Cancer Cells

**DOI:** 10.3390/medicina59030511

**Published:** 2023-03-06

**Authors:** Xiong Huang, Zhen Ma, Wei Qin

**Affiliations:** 1 Department of General Surgery, The Eighth People’s Hospital of Shanghai, Shanghai 200233, China; 2 Institute of Biomedical Sciences, Fudan University, Shanghai 200032, China; 3 Department of General Surgery, Huashan Hospital, Fudan University, Shanghai 200040, China

**Keywords:** Toll-like receptor, gastric cancer, miRNA, bioinformatics

## Abstract

*Background and Objectives*: To screen key miRNAs and their target genes related to Toll-like receptor (TLR) activation in gastric cancer (GC) cells and analyze them bioinformatically. *Materials and Methods*: Venn diagrams were obtained to screen miRNAs that were upregulated/downregulated in both GSE54129 and GSE164174. The miRTarBase database was used to predict the target genes of upregulated miRNAs. The differentially expressed genes in the regulatory network were analyzed. miR-16-5p expression in different tissue samples and the variations in the methylation states of four hub genes were measured. *Results*: We found that GSE54129 included 21 normal gastric tissues and 111 gastric cancer tissues, GSE164174 included 1417 normal gastric tissues and 1423 gastric cancer tissues. Venn diagram analysis results showed that compared with the control group, a total of 68 DEmiRNAs were upregulated in the GSE54129 and GSE164174 datasets, and no common downregulated DEmiRNAs were found. On further analysis of the GSE108345 dataset, we obtained the competing endogenous RNA (ceRNA) network associated with the activation of TLRs, and listed the top 10 lncRNA–miRNA–mRNA networks, including 10 miRNAs, 86 mRNA and 134 lncRNAs. Cytological HuBBA scores yielded a total of 1 miRNA, 16 mRNAs and 45 lncRNAs, of which miR-16-5p scored the highest as it was considered a key miRNA for TLR activation in GC cells, which are important in response against microorganisms. The results of Kyoto Encyclopedia of Genes and Genomes (KEGG) analysis showed that endocytosis, microRNAs in cancer and the PI3K-Akt signaling pathway are related to TLR signaling. The results of in vivo experiments indicated that miR-16-5p was highly expressed in gastric cancer cells and tissues. *Conclusions*: Hsa-miR-16-5p’s target genes mainly play a role by regulating the expression of four genes—MCL1, AP2B1, LAMB1, and RAB11FIP2. The findings provide a scientific basis for the development of immunotherapy for GC.

## 1. Introduction

Gastric cancer (GC) is a prevalent malignancy, ranking second among the causes of all cancer-related deaths worldwide [1]. More than 1 million new cases of GC and more than 800,000 deaths have been recorded globally in 2021 [2]. Among malignant tumors, China has the third-highest incidence and mortality rate associated with GC, with 42% of the total new cases [3]. GC is a multi-step and multi-factorial pathological process, wherein surgery remains the predominant treatment modality to date [4].

The advancements in bio-immunotherapeutics have increasingly provided options for the treatment of GC [5]. Recent advances in gene sequencing and other technologies have provided the theoretical basis for the diagnoses and treatment of GC at the molecular level. The pathogen-associated molecular pattern recognition receptors, Toll-like receptors (TLRs), exert crucial effects on natural immunity and secondary acquired immunity [6,7]. Additionally, TLRs perform vital functions in immune responses, inflammation, and tissue repair by activating the nuclear factor-kappa B (NF-κB), thereby mediating the levels of expression of several crucial cytokines, chemokines, and adhesion molecules. The stimulation of TLRs is also implicated in cellular proliferation and apoptosis [8,9]. Toll-like receptors (TLRs) belong to the large family of pattern recognition receptors (PRRs), and their activation leads to the induction of inflammatory cytokines, chemokines, antigen-presenting molecules, and co-stimulatory molecules. Different gene polymorphisms of these TLRs were found to be associated with gastric cancer. It has been confirmed that TLR1 and TLR10 gene polymorphisms were associated with a higher risk for gastric cancer in H. pylori-infected individuals [10]. TLR-based therapeutics are recognized as a promising approach in the treatment for GC [11]. However, the RNA transcriptional profiles and mechanisms of action underlying TLR activation in GC cells remain unclear. MicroRNA (miRNAs) is a general term for endogenous non-coding small RNAs; these are known to mediate expressions of different genes and participate in tumor development [12,13]. Therefore, miRNAs are promising targets for the diagnoses and/or treatment of numerous diseases [14,15,16]. 

In this study, we performed bioinformatics analyses, aimed at finding data to assist in further studies on immunotherapy and the mechanism underlying GC. We obtained the data of Venn diagrams to screen miRNAs that were upregulated/downregulated in both GSE54129 and GSE164174 [17]. The miRTarBase database was used to predict the target genes of upregulated miRNAs. The differentially expressed genes in the regulatory network were analyzed. miR-16-5p expression in different tissue samples and the variations in the methylation states of four hub genes were measured.

## 2. Materials and Methods

### 2.1. Gene Microarray Source and Differentially Expressed miRNAs and mRNAs

The gene microarray platforms for GC used in this study were GSE54129 (https://www.ncbi.nlm.nih.gov/geo/query/acc.cgi?acc=GSE54129, accessed on 16 January 2022) and GSE164174 (https://www.ncbi.nlm.nih.gov/geo/query/acc.cgi?acc=GSE164174, accessed on 21 January 2022); the application platform was the GPL21263 3D-Gene Human miRNA V21_1.0.0. GSE54129 included 21 normal gastric tissues and 111 gastric cancer tissues. The GSE164174 had the serum microRNA profiles of 2940 samples, which consist of 1423 gastric cancers, 1417 non-cancer controls, 50 esophageal cancers, and 50 colorectal cancers. In addition, GSE108345 (https://www.ncbi.nlm.nih.gov/geo/query/acc.cgi?acc=GSE108345, accessed on 25 January 2022), the gene microarray platform for the activation of TLRs in GC, was employed; the application platform was GPL570 (HG-U133_Plus _2) Affymetrix Human Genome U133 Plus 2.0 Array. 

After downloading the normalized expression matrices for the three datasets, first, the information on the gene ID of the probes was annotated. Next, the Fold Change was calculated using the comparative CT (2^−AACt^) method and the adjusted *p*-values were determined using *t*-tests. The screening criteria for the differentially expressed miRNAs (DEmiRNAs) were |log2Fold Change| > 2.0 and *p*-value < 0.05. The screening criteria of |log2FoldChange| > 1 and *p*-value < 0.05 were employed for differentially expressed mRNAs (DEmRNAs). Finally, volcano plots were generated to visualize the DEmiRNAss and DEmRNAs.

### 2.2. Venn Diagrams 

Venn diagrams were drawn using the VennDiagram package [18] to screen the miRNAs that were upregulated or downregulated in both GSE54129 and GSE164174.

### 2.3. The miRTarBase Database 

Target genes of the upregulated miRNAs were predicted using the miRTarBase database. The target genes were validated by luciferase labeling experiments and were considered as the differentially expressed genes (DEGs) in GSE108345. Similarly, target long non-coding RNAs (lncRNAs) of upregulated miRNAs were predicted. LncRNA-miRNA–mRNA regulatory networks were subsequently generated using the Cytoscape software (The Cytoscape Consortium, San Diego, CA, USA) and key subnetworks were identified using the cytoHubba plug-in (The Cytoscape Consortium, San Diego, CA, USA). The MCC calculation method was used to identify the parameters of the subnetworks, with the display number set to the top 10.

### 2.4. Kyoto Encyclopedia of Genes and Genomes (KEGG) Enrichment Analysis

The KEGG database is among the most commonly used bioinformatics databases, of which the KEGG pathway database is its core, using R language (https://www.r-project.org/, accessed on 25 January 2022) related data packages, based on target genes related to miR-16-5p, key signaling pathways were obtained through KEGG enrichment analysis.

### 2.5. Reverse Transcription-Polymerase Chain Reaction (qRT–PCR) 

qRT–PCR was performed to evaluate the level of miR-16-5p expression in different tissue samples from patients of the Department of General Surgery, the Eighth People’s Hospital of Shanghai. Briefly, total RNA was extracted from the tissues by using TRIzol reagent (Invitrogen, Carlsbad, CA, USA) following the manufacturer’s instructions. The cDNA was synthesized using RT of the Takara Reverse Transcription Kit (Takara Bio Inc., Otsu, Japan). Quantified analysis of miR-16-5p and U6 microRNA levels was conducted using the SYBR Green Master Mixes (Thermo Fisher Scientific, Shanghai, China) on 7300 Real-Time PCR System (Applied Biosystems, Waltham, MA, USA). The reaction was performed at 94 °C for 1 min, 55 °C for 1 min, and 72 °C for 1 min (35 cycles) and the extension was for 5 min at 72 °C. The level of miR-16-5p expression was calculated by the 2^−△△Ct^ method. The primers were designed as follows: firstly, the miRBase database was used to analyze the sequence of the miRNA. If the predicted melting temperatures (Tm) between the primers and the target sequence is high, then shortening the length of the primer can be used to increase specificity. If the predicted Tm between the MSP and the target sequence is low, the cDNA was synthesized using the miQPCR approach by extending the 3′-end of the primers. The unspecific amplification products were prevented by ensuring that the primer will not anneal to linker sequence. The sequences of the related primers used were listed as follows: F: 5′-GTGCCAGAAACCGTTGAATC-3′; R: 5′-TTGTGTTTCTGTGCCTCGTT-3′; U6: F: 5′-CAACAGGCTCGTGAAAGACC-3, R: 5′-GTTCGTCAACCTAGCGCAG-3. The collection and usage of these tissues were approved by the institutional ethic committee and the written informed consents were obtained from the donors.

### 2.6. Fluorescence In Situ Hybridization

The expression level of miR-16-5p in GC tissues from patients of the Department of General Surgery, the Eighth People’s Hospital of Shanghai was evaluated by fluorescence in situ hybridization (FISH) using an alexa Fluor 488-labeled miR-16-5p probe (RiboBio, Guangzhou, China). The probe signals were determined with the FISH Kit (BersinBio, Guangzhou, China) according to the manufacturer’s guidelines. In brief, the slides of human GC and corresponding control tissues were incubated at 37 °C for 10 min with Proteinase K reagent (Qiagen, Germantown, MD, USA). Then, 50 μL of hybridization mix (including RNA probes) was applied to the slides, and each section was covered with a sterile cover slip. After hybridization for 1 h, the slides were washed 5 times in saline sodium citrate buffer and blocked for 15 min with blocking solution in a humidified chamber. After that, the slides were incubated with the anti-FAM/CY3 for 60 min. The freshly prepared AP substrate was applied to the sections and then was incubated for 2 h at 30 °C. Then, we stopped the reaction by using KTBT buffer (50 mM Tris–HCl, 150 mM NaCl and 10 mM KCl). Finally, 300 μL 4′,6-Diamidine-2′-phenylindole dihydrochloride (DAPI) (Beyotime, Shanghai, China) was added to the sections. Images were acquired using a fluorescence microscope (BX53, Olympus, Tokyo, Japan). The collection and usage of these tissues were approved by the institutional ethic committee and the written informed consents were obtained from the donors.

### 2.7. Statistical Analysis

The SPSS 25.0 software (SPSS, Inc., Chicago, IL, USA) was used for all statistical evaluations. An independent-samples *t*-test was used for comparison between two groups of continuous variables, while the chi-square test was employed for comparison between two groups with categorical variables. The significance level was defined at *p* < 0.05.

## 3. Results

### 3.1. Data Collection and Difference Analysis Results

Two miRNA core datasets were collected from the gene expression omnibus (GEO) database: GSE54129 and GSE164174. After the expression data were standardized, we obtained the hub genes and key clusters of DEmiRNAs target genes between control GC and TLR-activated GC tissues. The gene chip data of GC and the gene chip data related to TLR activation in GC are shown in Table 1 and Table 2. It can be seen that there were 111 gastric cancer samples in GSE54129 and 21 in the control group, 1417 gastric cancer samples in GSE164174 and 487 in the control group (Table 1). There were nine TLR-activated samples and nine TLR-unactivated samples in GSE108345 (Table 2).

**Figure 1:**
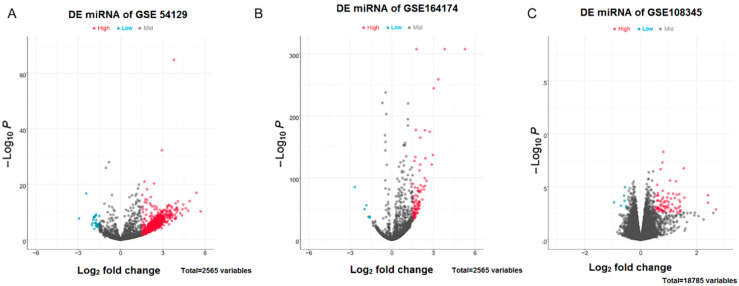
Volcano plots for DEmiRNAs. (**A**) DEmiRNAs in the GSE54129 dataset; (**B**) DEmiRNAs in the GSE164174 dataset; (**C**) DEmiRNAs in the GSE108345 dataset. The horizontal coordinates indicate log2FoldChange, while vertical coordinates indicate the −log10P-value. Blue color indicates significantly downregulated DEmiRNAs, whereas red color indicates significantly upregulated DEmiRNAs.

**Figure 2:**
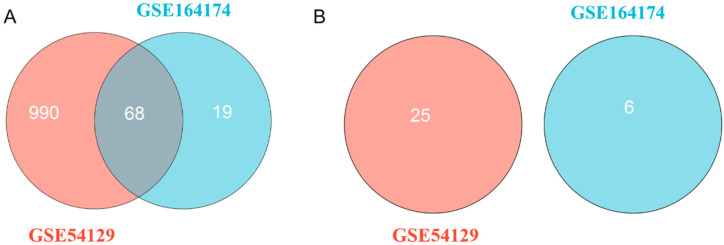
Venn diagram for DEmiRNAs. (**A**) A total of 68 upregulated DEmiRNAs in both the GSE54129 and GSE164174 datasets; (**B**) no DEmiRNA is downregulated commonly in both the GSE54129 and GSE164174 datasets.

According to the results of difference analysis, blue indicates that the DEmiRNA is significantly downregulated in the volcano diagram, while red indicates that the DEmiRNA is significantly upregulated. Further Venn diagram analysis results show that a total of 68 DEmiRNAs in GSE54129 and GSE164174 datasets are upregulated compared with the control group. However, DEmiRNA downregulation was generally absent in both the GSE54129 and GSE164174 datasets (Figure 1 and Figure 2).

### 3.2. Results of Co-Expression Network Analysis

**Figure 3:**
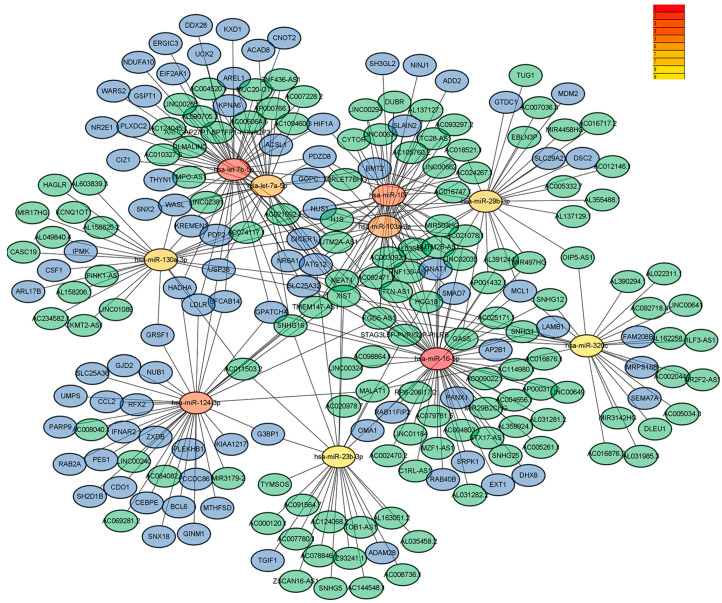
Results of pathway enrichment analysis (multiple miRNAs).

**Figure 4:**
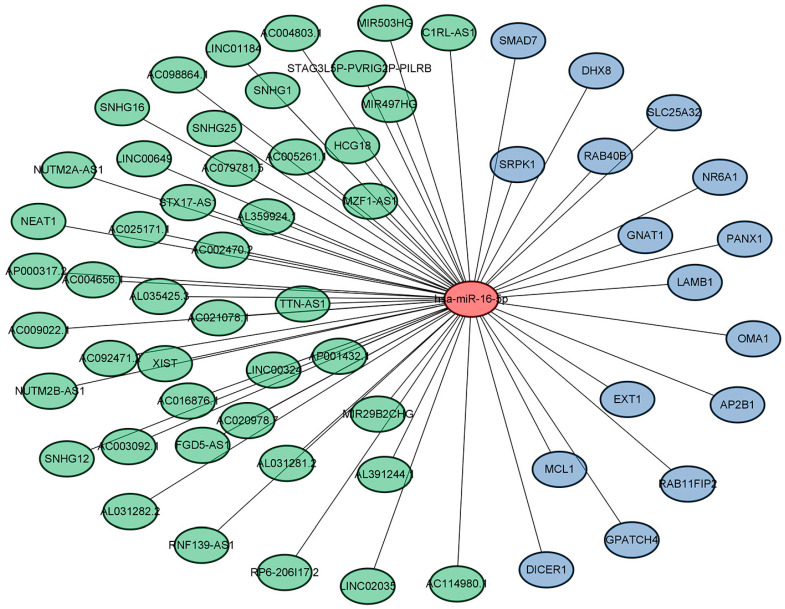
Results of pathway enrichment analysis (miR-16-5p).

Next, analysis of the GSE108345 dataset yielded a competitive endogenous RNA (ceRNA) network associated with TLR activation (Figure 3 and Figure 4). The top 10 miRNA-regulated lncRNA–miRNA–mRNA networks were ranked according to the cytoHubba score. MiRNAs are represented by gradient red nodes according to their cytological HUBBA score, lncRNAs are represented by green nodes, and mRNAs are represented by blue nodes. In total, there are 10 miRNAs, 86 mRNAs, and 134 lncRNAs. A total of 1 miRNA, 16 mRNAs and 45 lncRNAs were generated by Hub Objects Analyzer (Hubba) score of cytology, among which miR-16-5p had the highest score. Hsa-miR-16-5p was found to be a key miRNA for TLR activation in GC cells. This may affect the biological function and endocytosis of the PI3K/AKT signaling pathway by regulating four hub genes, namely myeloid cell leukemia 1 (*MCL1*), adaptor-related protein complex 2 subunit beta (*AP2B1*), laminin subunit beta 1 (*LAMB1*) and RAB11 family-interacting protein 2 (*RAB11FIP2*), and eight lncRNAs (Table 3 and Table 4).

**Figure 5:**
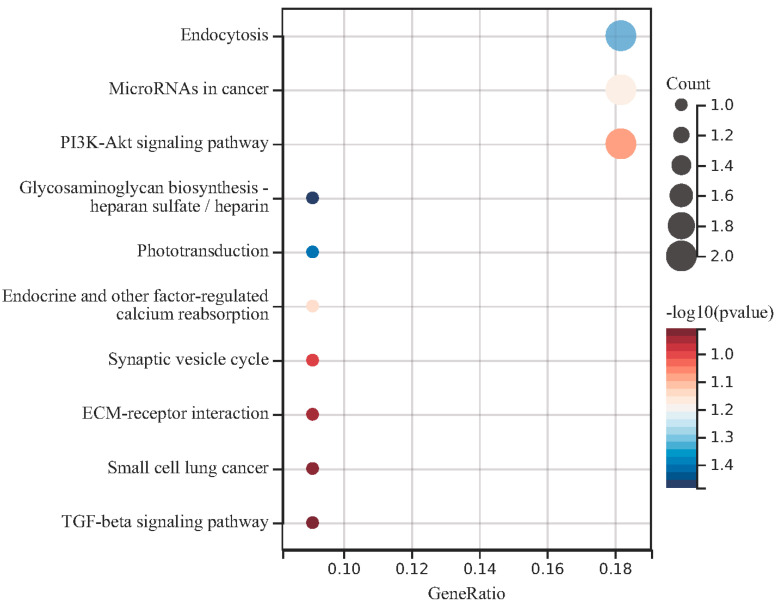
Enrichment analysis for the target genes in the regulatory networks. The KEGG enrichment analysis results were sorted according to the *p* value, and the darker red color indicated that the smaller the *p* value, the higher the enrichment degree.

The KEGG enrichment analysis results of the DEGs in the regulatory network using the cluster analyzer package are shown in Figure 5. The results of KEGG analysis showed that endocytosis, microRNAs in cancer and the PI3K-Akt signaling pathway are related to TLR signaling (*p* < 0.05).

### 3.3. Expression Results of miR-16-5p in Gastric Cancer Cells and Tissues

**Figure 6:**
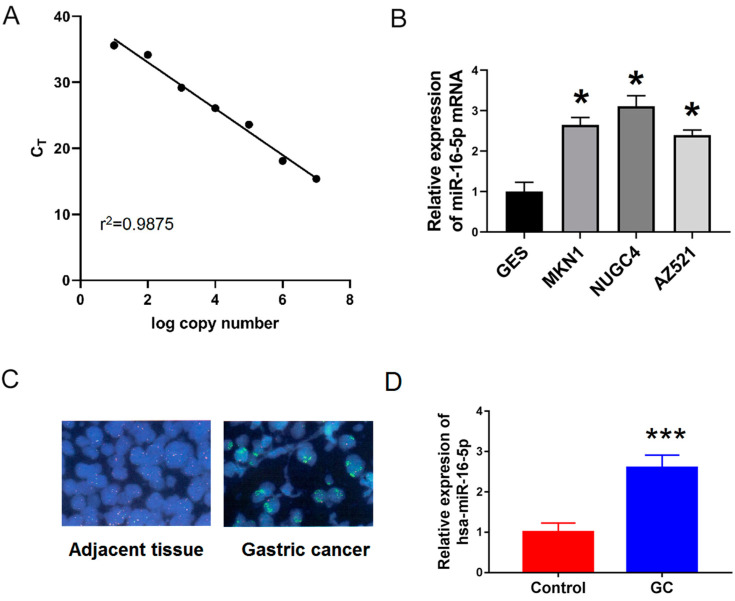
miR-16-5p was upregulated in GC cells and tissues. (**A**) Standard curves were constructed for miR-16-5p using qRT-PCR assay. (**B**) qRT-PCR was used to examine the relative levels of miR-16-5p in human MKN1, NUGC4 and AZ521) compared with that in GES-1 cells. (**C**,**D**) Fluorescence in situ hybridization (FISH) and qRT-PCR were used to examine the expression of miR-16-5p in 25 pair’s GC and adjacent tissues. * *p* < 0.05 vs. GES. *** *p* < 0.001 vs. control.

The expression of miR-16-5p was measured in these GC cells in different GC cells (MKN1, NUGC4 and AZ521). As shown in Figure 6B, miR-16-5p was significantly upregulated in GC cells (including MKN1, NUGC4, and AZ521) compared with gastric epithelium cells (GES-1).

To further confirm this, we used FISH in situ hybridization and qRT-PCR to detect the expression of miR-16-5p in 25 pairs of gastric cancer and adjacent tissues. As shown in Figure 6B,C, the expression of miR-16-5p in GC tissues was significantly higher than that in adjacent tissues. Together, all these results suggest that miR-16-5p is a potential tumor promoter in GC progression.

## 4. Discussion

The rapid development of high-throughput sequencing methods has facilitated a deeper understanding of the molecular mechanisms underlying GC, including altered pathogenesis, invasion, and metastases. However, immune-related studies are scarcely reported [19,20,21]. TLRs, key regulators of the innate immune responses, are dysregulated in many inflammation-related malignancies, including GC [22,23,24]. However, the identity of the specific TLRs and their molecular targets contributing to the pathogenesis of human GC remains unclear [25,26]. Previous studies have identified the significance of TLR expressions in human GC cells. Levels of mRNA and protein expression of TLRs are reportedly elevated in tumor tissues of more than 50% of patients with GC, and TLRs are also significantly expressed in human GC cell lines [27,28,29]. Huang et al. revealed that miR-155-mediated inhibition of DEPTOR (an inhibitor of mTOR) with secondary activation of mTOR was a potential marker for resistance to Helicobacter pylori eradication therapy in Gastric diffuse large B-cell lymphoma. In contrast, Toll-like receptor 5 (TLR5) was shown to be a potential marker for sensitivity to Helicobacter pylori eradication therapy [30]. In another study, it was demonstrated that miR-198 could induce apoptosis and inhibit the proliferation, migration, and invasion of GC cells through downregulating TLR4 expression [31]. Yan et al. found out that hsa-miR-335 was involved in regulating target genes in several oncogenic signal-pathways in gastric cancer development, including mTOR, Toll-like receptor and focal adhesion [32]. All these studies suggested that miRNAs were closely related to the prognosis of GC patients. In this study, we statistically analyzed the hub genes of miRNAs associated with TLR activation in GC using sequencing technology, to provide support for the diagnosis and treatment of GC at the molecular level. Moreover, TLRs are important in response against microorganisms. It is possible that miRNAs could also play a role in the body response to infections and be used to treat against microorganisms.

In this study, a series of bioinformatics analyses were performed using the data downloaded from the gene expression omnibus (GEO) database. We obtained the hub genes and key clusters of the target genes of DEmiRNAs between the control-GC and TLR-activated GC tissues. Our findings showed that a total of 68 DEmiRNAs were upregulated in both the GSE54129 and GSE164174 datasets; however, no DEmiRNA was downregulated commonly in the two datasets. The analyses of the GSE108345 dataset yielded a ceRNA network associated with the activation of TLRs. The lncRNA–miRNA–mRNA network regulated by the top 10 miRNAs ranked according to their cytoHubba scores resulted in a total of 10 miRNAs, 86 mRNAs, and 134 lncRNAs. miR-16-5p, having the highest cytoHubba score was associated with a total of 1 miRNA, 16 mRNAs, and 45 lncRNAs. The expression of miR-16-5p was significantly altered in the sera of patients with non-small-cell carcinoma, hepatocellular carcinoma, and GC [33]. In a previous study, miR-16-5p showed a sensitivity of 0.88 and a specificity of 0.86 for the diagnosis of non-small-cell lung cancer [34]. The sensitivity of miR-16-5p for diagnosing liver cancer was found to be 0.721 with a specificity of 0.888 [35]. In addition, the sensitivity of miR-16-5p for diagnosing GC was 0.79 and the specificity was 0.78 [36]. These results indicate that miR-16-5p could be a potential diagnostic biomarker for certain specific tumors [37]. Recent studies suggest that miR-16-5p expression is upregulated in the sera of patients with GC [38,39], consistent with the findings of the present study. Additionally, miR-16-5p plays a crucial function in the proliferation, invasion, and metastases of different tumor cells [40]. miR-16-5p suppresses the proliferation and invasion of breast cancer cells and promotes their apoptosis by targeting the vascular endothelial growth factor-A. Additionally, miR-16-5p inhibits the invasion and migration of hepatocellular carcinoma cells by directly targeting the insulin-like growth factor 1 receptor. In a study on colorectal cancer, miR-16-5p was implicated as an oncogenic agent which functioned via the VEGFA/VEGFR1/Akt signaling axis. These results indicate that miR-16-5p is a key factor for the progression of numerous tumors [41]. miR-16-5p is markedly upregulated in both sera and cancerous tissues of patients with GC and may serve as a biomarker for GC diagnosis. miR-16-5p may influence the progression of GC by regulating translation, binding of proteins, cell adhesion, and plays a crucial role in the immunotherapy of GC through multiple tumor-related signaling pathways [42]. In particular, miR-16-5p can act on the TLR pathway. A previous study has shown that the Starbase database predicted that miR-16-5p had a binding site of TLR4 [43]. The miR-16-5p mimics downregulated the expression of TLR4, while TLR4 overexpression restored the TLR4 expression that was reduced by miR-16-5p overexpression [43].

Based on the findings of this study, we inferred that miR-16-5p could affect biological functions such as the PI3K-Akt signaling pathway and endocytosis by regulating four hub genes, namely *MCL1, AP2B1, LAMB1,* and *RAB11FIP2*, and eight lncRNAs. The *MCL1* gene is located on chromosome lq21 and encodes a protein of 42,000D with 350 amino acid residues. *MCL1*, significantly expressed in several normal tissues, is essential for maintaining the maturation and differentiation of different cell types, including the T cells, B cells, and macrophages. Upregulation of *MCL1* expression suggests tumorigenesis, with greater than 85% of *MCL1* transgenic mice developing B-cell lymphoma within two years [44]. *MCL1* is highly expressed in different malignancies and reduced *MCL1* expression in cells promotes apoptosis, inhibits cancer cell proliferation, along with cell cycle arrest [45]. Herein, *MCL1* was found to be significantly expressed in GC tissues. In the *AP2B1* gene, AP-2 sorts cargo proteins, assembles and disassembles clathrins, and recruits accessory proteins for the formation of clathrin-coated vesicles [46]. The full-length sequence of the *AP2B1* gene is 3176 bp long. It exerts physiological and pathological effects by regulating cellular and protein transport [47]. LAMB is the most abundant non-collagenous glycoprotein in the basilar membrane. To date, over 14 laminin isoforms have been reported, each having a specific tissue distribution and function. The YIGSR peptide of LAMB1 binds to the 67 kDa laminin receptor (LAMR) and their interaction promotes cellular adhesion. A previous study reports that LAMB1 may also be involved in cellular angiogenesis, proliferation, invasion, and migration [48]. LAMB1 overexpression is associated with the metastases and progression of multiple malignant tumors. Therefore, overexpression of LAMB1 and its receptor LAMR may play a crucial role in the progress of GC. LAMB1 is upregulated in hepatocellular carcinoma tissues and its co-expression with keratin-19 leads to poor prognosis and shortened survival in these patients. Changes in LAMB1 levels in the sera of patients with colorectal cancer may therefore be a novel putative diagnostic biomarker for colorectal cancer. Rab11FIP2 is a member of the Rab11-interacting proteins, with a structure consisting of a Rab11-binding domain at the C-terminus and a C2 phospholipid-binding domain near the N-terminus. Rab11FIP2 interacts with MYO5B and Rab11 to regulate circulating vesicle transport and plasma membrane recycling, both of which play crucial roles in regulating cellular biological behaviors [49]. Rab11FIP2 can be induced by the hypoxia-inducible factor-alpha, thereby increasing its expression, which in turn promotes the metastases of GC cells through epithelial-mesenchymal transition and activation of the Akt signaling cascade [50]. Overexpression of Rab11-FIP2 promotes metastasis of colorectal cancer cells through the PI3K/Akt/ Matrix Metalloproteinase 7 (MMP7) pathway, thus affecting the prognoses of these patients [51]. Given the high economic cost of current technologies such as gene microarray and high-throughput sequencing, herein, we screened information from the GEO database for secondary analysis. Therefore, the results of this study may show some deviation from the real situation. As a result, the accuracy and reliability of the above analyses need further experimental verification in the future.

## 5. Conclusions

In conclusion, the activation of TLRs in GC cells suggested that immune responses exerted crucial effects on the progression of GC. The underlying mechanism is likely regulated by the differentially expressed Hsa-miR-16-5p gene. Additionally, hsa-miR-16-5p could specifically regulate four hub target genes (*MCL1, AP2B1, LAMB1,* and *RAB11FIP2*), and their methylation states, thereby predicted to affect the prognoses of patients with GC. In particular, we used FISH in situ hybridization and qRT-PCR to detect the expression of miR-16-5p in gastric cancer and found out that the expression of miR-16-5p in GC tissues was significantly increased. It may serve as a predictive indicator for patient’s disease outcome. Moreover, the target genes (*MCL1, AP2B1, LAMB1,* and *RAB11FIP2*), and their methylation states can be considered as treatment targets of patients with GC, and high-risk patients would accept additional therapeutic intervention.

## Figures and Tables

**Table 1:** Gene microarray data for GC.

Dataset	GC	Control	Dataset Type
GSE54129	111	21	miRNA expression matrix
GSE164174	1417	487	miRNA expression matrix

Gastric cancer (GC).

**Table 2:** Gene microarray data related to activation of TLRs in GC.

Dataset	TLR Activated	TLR Unactivated	Dataset Type
GSE108345	9	9	mRNA expression matrix

Toll-like receptor (TLR); gastric cancer (GC).

**Table 3:** Hub genes and their corresponding enriched pathways in the key subnetwork.

Target miRNA	Gene Symbol	Enriched Pathways
hsa-miR-16-5p	*MCL1*	PI3K-Akt signaling pathway
hsa-miR-16-5p	*AP2B1*	Endocytosis
hsa-miR-16-5p	*LAMB1*	PI3K-Akt signaling pathway
hsa-miR-16-5p	*RAB11FIP2*	Endocytosis

AP-2 complex subunit beta (*AP2B1*), myeloid cell leukemia-1(*MCL1*), recombinant laminin beta 1 (*LAMb1*), RAB11 family-interacting protein 2 (*RAB11FIP2*), phosphoinositide-3 kinase (*PI3K*), and protein kinase B (*Akt*).

**Table 4:** lncRNAs in the key subnetworks sorted according to the node scores (from highest to lowest).

Rank	lncRNA	Degree
1	NUS1	30.611
2	PDP2	27.012
3	WASL	26.359
4	MALAT1	22.366
5	KPNA6	20.864
6	EFCAB14	19.43
7	BMT2	18.694
8	AC011503.2	16.942

Long non-coding RNA (lncRNA), Nogo-B receptor (NUS1), pyruvate dehydrogenase phosphatase isoenzyme 2 (PDP2), Wiskott–Aldrich syndrome-like (WASL), metastasis-associated lung adenocarcinoma transcript 1 (MALAT1), karyopherin alpha 6 (KPNA6), EF-hand calcium-binding domain-containing protein 14 (EFCAB14), and bone marrow stromal antigen 2 (BMT2).

## Data Availability

The datasets used and/or analyzed for this study are available from the corresponding author upon reasonable request.
